# The olfactory test established by Henkin: is it reliable and does it correlate to established psychophysical tests?

**DOI:** 10.1007/s00405-024-08900-w

**Published:** 2024-08-23

**Authors:** Anna Kristina Hernandez, Irina Käb, Thomas Hummel

**Affiliations:** 1 Smell and Taste Clinic, Department of Otorhinolaryngology, Faculty of Medicine Carl Gustav Carus, Technische Universität Dresden, Fetscherstraße 74, 01307 Dresden, Germany; 2 Department of Otolaryngology – Head and Neck Surgery, Asian Hospital and Medical Center, Muntinlupa, Philippines

**Keywords:** Psychophysical, Olfactory, Smell, Henkin, Test

## Abstract

**Purpose:**

This pilot study aimed to determine whether the Henkin olfactory test discriminates between the olfactory function of patients with olfactory loss and healthy individuals, and whether the Henkin test is correlated with a validated psychophysical olfactory test.

**Methods:**

Participants underwent olfactory testing using the modified Henkin test (including detection [DT] and recognition [RT] thresholds, magnitude estimation [ME], and hedonic ratings [H], for four different odors) and the extended “Sniffin’ Sticks” test battery (odor detection, discrimination, identification).

**Results:**

Forty-four individuals (28 women, aged 19–81 years, mean: 39 years) were included. Twenty-three were healthy (mean age: 38 years) and 21 had olfactory dysfunction (OD) (mean age: 40 years). OD patients had worse mean DT, lower composite RT, and lower ME ratings. Mean and individual odor H ratings were not significantly different between the groups. Most individuals were classified as hyposmic when using the prescribed classification by Henkin, with only very few satisfying the stringent criteria of anosmia and normosmia. Modified Henkin subtests were all positively correlated with each other and with the “Sniffin’ Sticks”, except for Unpleasant Mean H which was only correlated with Pleasant Mean H; and Pleasant mean H which was not correlated with mean DT scores.

**Conclusion:**

The Henkin test is able to separate between OD patients and controls. However, modifications to the conduct of this test may be required. Studies that used this test should be carefully interpreted.

**Level of evidence:**

3.

## Introduction

The olfactory test developed by Henkin has been cited in literature from as early as 1967 [1]. This article described only the threshold subtests but later publications [2, 3] included a psychophysical test with 4 subtests: detection threshold (DT) [1]; recognition threshold (RT) [1]; magnitude estimation (ME) [4]; and hedonic value/rating (H) [3]. These four subtests determined sensory receptor functioning (DT), the connectivity between functioning sensory receptors and the brain (RT), the number of functioning sensory receptors (ME), and the presence or absence of distortions in sensory information processing (H) [5]. 

This test was the basis for several frequently cited studies published in renowned journals (e.g., [3, 6–9]), particularly on Theophylline as a treatment for olfactory dysfunction (OD) [3, 6, 7, 9]. However, these articles often only cited previous publications for the methodologic details [3, 6, 7, 10], which at times referred to other previous publications as well, with conflicting methodologic details. Only the first article mentioning DT and RT [1] completely described these concepts and this article was cited in another article without the title of the study [11], making the subsequent search for these references challenging. To the best of our knowledge, no previous studies have described the validity of the Henkin olfactory test against a patient group with established anosmia or other validated psychophysical olfactory tests. Due to this, questions remain about the conclusions drawn from studies using this method of testing.

This pilot study aims to determine whether the Henkin olfactory test discriminates between patients with olfactory loss and healthy individuals; and whether the different subtests of the Henkin test are correlated with a validated psychophysical olfactory test.

## Methods

This study included adults (≥ 18 years old), who consulted at the Smell and Taste Clinic, Department of Otorhinolaryngology, University Hospital Dresden for COVID-19 and idiopathic OD. Patients with other major diseases that could affect olfactory function (i.e., Parkinson’s disease, diabetes, epilepsy) were excluded. The study design was approved by the ethics committee at the University Clinic Dresden (application number EK 156052012). The control group was composed of healthy volunteers without olfaction-related complaints. A standardized structured history was taken and participants were tested using both the Henkin test and the “Sniffin’ Sticks” (Burghart Messtechnik, Holm, Germany). For technical reasons, the “Sniffin’ Sticks” test was always performed first. The “Sniffin’ Sticks” (SS) test included assessments for odor threshold (T), discrimination (D), and identification (I) (for more details, see [12]).

### Henkin Test

#### Detection and recognition threshold

DT was performed using a three-alternative forced choice, staircase, sniff technique [2] with four odorants (Thiophene [Th, “petroleum-like”], Pyridine [Pyr, “fish”], Nitrobenzene [NB, “bitter almond”], and Amyl Acetate [AA, “banana”] [3] diluted in paraffin oil). Each odorant had 11 concentrations (from Pure, 10^0^ to 10^− 9^, Table 1), diluted at a ratio of 1:10 with the pure odorant as the highest concentration. Testing began at concentration step (CS) 5. Participants were blindfolded and randomly presented with a set of 3 test solutions in 50 ml dark glass bottles held at 2 cm from the nostrils [1]. One contained the odorant, while two contained only solvent [4]. Participants were tasked to describe which of the three bottles had a different odor. If the participant gave the correct response for CS 5, then testing proceeded to the next lower concentration until the odorant was incorrectly identified. This produced a reversal of the staircase until the odorant was correctly identified twice. This was noted as the final threshold, provided that the next higher concentration was correctly identified [4].

**Table 1:** Smell test bottle dilutions

Odorant	Thiophene (Th)	Pyridine (Pyr)	Nitrobenzene (NB)	Amyl Acetate (AA)
Pure + Paraffin Oil (PO) (Highest Concentration)	Pure + PO	Pure + PO	Pure + PO	Pure + PO
10^0^	Th-0	PYR-0	NB-0	AA-0
10^− 1^	Th-1	PYR-1	NB-1	AA-1
10^− 2^	Th-2	PYR-2	NB-2	AA-2
10^− 3^	Th-3	PYR-3	NB-3	AA-3
10^− 4^	Th-4	PYR-4	NB-4	AA-4
10^− 5^	Th-5	PYR-5	NB-5	AA-5
10^− 6^	Th-6	PYR-6	NB-6	AA-6
10^− 7^	Th-7	PYR-7	NB-7	AA-7
10^− 8^	Th-8	PYR-8	NB-8	AA-8
10^− 9^(Lowest Concentration)	Th-9	PYR-9	NB-9	AA-9

RT was performed after the DT. Based on previous publications, participants should be asked “Of the different odorant(s), what is its character [pungent, putrid, sweet, minty, flowery, fruity, sweaty or some other common description(s)]” [4] (Table 2). The lowest concentration in which the odorant was correctly identified becomes the RT. However, modifications to testing reflected, instead, whether participants were able to correctly or incorrectly identify the odor based from a list of descriptors (Table 3) at or above the level of the normal RT (CS 2). A composite RT score comprised of the sum of correct identifications (maximum: 4) was also included in the analysis.

**Table 2:** Acceptable responses to recognition threshold test for each odor

**Thiophene (Th)**	**Pyridine (Pyr)**
• Pungent	• Putrid
• Gasoline	• Pungent
• Natural Gas	• Dead Fish
• Sulfur	• Onion
• Refinery Smell	• Garlic
• Rotten Eggs	
• Garlic	
• Onion	
• Rubbery	
• Oil	
**Nitrobenzene (NB)**	**Amyl Acetate (AA)**
• Almond	• Banana
• Bitter Almond	• Fruit
• Flowery	• Nail Polish
• Shoe Polish	• Nail Polish Remover
• Nutty	• Paint
• Cinnamon	• Paint Thinner
• Fruity and Minty are less desirable but can be accepted	• Sweet
	• Minty and Flowery are less desirable but can be accepted

**Table 3:** Classification of smell loss (modified from [3, 4])

Test	Normal	Hyposmia			Anosmia
		Type 1	Type 2	Type 3	
DT	DT ≥ 5^*^	0 < DT < 5	0 < DT < 5	DT ≥ 5	0
RT	RT ≥ 2^+^	0	0 < RT < 2	RT ≥ 2	0
ME	ME ≥ 48%	0	0 < ME < 48%	0 < ME < 48%	0
H	H = normal	0	0 < H < normal	0 < H < normal	0

DT: detection threshold; RT: recognition threshold; ME: magnitude estimation; H: hedonic ratings; For DT and RT: higher score refers to lower odor concentration; ^*^Normal DT: correctly detected at 10^− 5^ (score = 5) or lower concentration (score > 5); ^+^Normal RT: correctly identified at 10^− 2^ (score = 2) or lower concentration (score > 2)

In a 2009 study by Henkin et al., results for thresholds were “converted into bottle units as previously described” and reported as “the mean ± standard error of the mean (SEM) of the correct responses [concentrations (in moles/liter)] for each odor for each treatment group” [3]. However, for easier comparability with the “Sniffin’ Sticks”, CSs were re-coded into whole numbers (from 0 [highest concentration, pure] to 10 [lowest concentration, 10^− 9^).

#### Magnitude estimation and hedonics

Historically, ME was reported in percent and reflected “the means for all correct responses using data for the 4 highest odor concentrations presented (from 10^− 2^ [CS 2] to pure) [3, 4], where the normal ME was ≥ 50% [4]; while H was determined by asking: “do you find this described smell pleasant (+ 1 to + 100), unpleasant (-1 to -100), or neutral (0).” [3] Th and Pyr are usually unpleasant (negative H); while NB and AA are usually pleasant (positive H). If H responses to usually positive odorants are negative, then the patient is considered to exhibit parosmia [4] (term used in original reference was dysosmia [13]). The means ± SEMs were again used to report the results. ME was modified as the mean of intensity ratings for CS2 or higher (depending on the DT identified) using a scale from 1 to 10 (highest), with a score of ≥ 4.8 regarded as normal [3]; and H was modified as a rating between - 1 to - 5 (unpleasant), + 1 to + 5 (pleasant), and 0 (neutral).

Historically, smell loss was classified by the Henkin test according to Table 3. Comparison of frequency of individuals classified as having smell loss based on both tests were determined.

### Statistical analyses

Statistical analyses were performed using SPSS ver. 28.0 (IBM SPSS Statistics for Windows, Vs. 28.0; IBM Corp., Armonk, NY, USA). T-tests and Pearson’s r correlations were performed for continuous variables (mean DT, composite RT, mean ME, unpleasant mean H, pleasant mean H, and TDI scores), Chi-square test and Fisher’s Exact Test were done for nominal variables (Group, Classification), and a p-value of < 0.05 was considered significant. Post-hoc power analysis was performed using G*power (Version 3.1.9.7, https://www.psychologie.hhu.de/arbeitsgruppen/allgemeine-psychologie-und-arbeitspsychologie/gpower; Germany).

## Results

A total of 28 women and 16 men, aged 19–81 years (mean: 39) participated in the study. At baseline, age and gender were not significantly different between groups. Twenty-three were healthy and 21 had OD from COVID-19 (*n* = 12), idiopathic causes (*n* = 7), and others (*n* = 2).

When using the Henkin classification (see Table 3), only 8 individuals satisfied the predetermined criteria (Table 4). The Henkin test tended to cluster most individuals as having hyposmia, given the stringent requirement of absolute absence of perception in all 4 subtests prior to being classified as having anosmia. Hyposmia was overdiagnosed in 17 out of 23 controls (74%), while anosmia was underdiagnosed in 9 (43%) and hyposmia was underdiagnosed in 1 out of 21 (5%) patients. Although the Henkin test was able to correctly categorize 17 out of 44 individuals (39%), group (OD patients, controls) was not significantly associated with smell loss classification on chi-square test. In comparison, group was significantly associated with smell loss classification (Fisher’s exact = 25.26, *p* < 0.001) for the SS test. Only one healthy individual was categorized as hyposmic, with a TDI score of 30.5 (upper limit of hyposmia) [12]. Day-to-day olfactory fluctuations may occur in an individual’s score [14–16]. Since the score is at the borderline, it may be possible that this individual scores within the normal range on repeat testing.

**Table 4:** Frequency of olfactory loss based on the modified Henkin test

Variables		Normal	Hyposmia			*Presumed Hyposmia*												Anosmia
			Type 1	Type 2	Type 3													
Test	DT	2	1	1	2	2	2	2	2	2	2	2	2	2	1	1	1	0
	RT	2	0	1	2	2	1	1	1	1	1	1	0	0	1	1	0	0
	ME	2	0	1	1	2	2	2	2	1	1	1	1	1	2	1	1	0
	H	2	0	1	1	1	2	1	0	2	1	0	2	1	1	2	1	0
Group	Controls	**5**	**0**	**0**	**0**	**2**	**4**	**11**	**1**	**0**	**0**	**0**	**0**	**0**	**0**	**0**	**0**	**0**
	OD patients	**1**	**0**	**2**	**0**	**1**	**1**	**6**	**0**	**1**	**1**	**1**	**1**	**1**	**1**	**2**	**2**	**0**
	Total n	6*	**0**	2*	**0**	**3**	**5**	**17**	**1**	**1**	**1**	**1**	**1**	**1**	**1**	**2**	**2**	**0**

DT: detection threshold; RT: recognition threshold; ME: magnitude estimation; H: hedonic ratings; n: number of individuals; OD: olfactory dysfunction; Values under the row heading “Test” reflect the scoring pattern based on Table 3, where the score of 2 corresponds to: DT ≥ 5 OR RT: composite RT = 4 OR ME ≥ 4.8 OR H: correct [pleasant rated +, unpleasant rated -] for all odors; a score of 1 corresponds to: 0 < DT < 5 OR 0 < composite RT < 4 OR 0 < ME < 4.8 OR H: correct for 1 group, incorrect or neutral for the other; and a score of 0 corresponds to H: neutral for all odors. Values under the row heading “Group” reflect the frequency of participants having a specific pattern of scores, as they occur in controls, OD patients, and in the total sample. “Presumed Hyposmia” refers to the different patterns of responses that do not fit with hyposmia types specified in previous publication (Types 1, 2, and 3) but also do not satisfy the criteria for normosmia or anosmia; *Only 8 individuals had score patterns corresponding to the predetermined criteria based on Table 3

The mean DT (t_15.11_=5.14, *p* < 0.001), mean ME (t_15.38_=4.55, *p* < 0.001), were significantly worse in patients compared to controls (Table 3). Unpleasant and pleasant mean Hs were not different between the groups. We additionally performed a power analysis and determined that when using t-tests to distinguish between patients and controls, Mean DT, Composite RT, and Mean ME all had a power > 0.90. The power for the analysis of Unpleasant H was 0.76, while that of Pleasant H was 0.55. This showed a large overlap in responses between the two groups. Given that the responses for hedonics had to be divided into 2 groups (pleasant and unpleasant, further cutting down the sample by half), this rendered the analysis for Hs to have a lower power compared to the other subtests. Healthy individuals also had a higher composite RT score than those with OD (t_33.70_=3.63, *p* < 0.001).

**Fig. 1:**
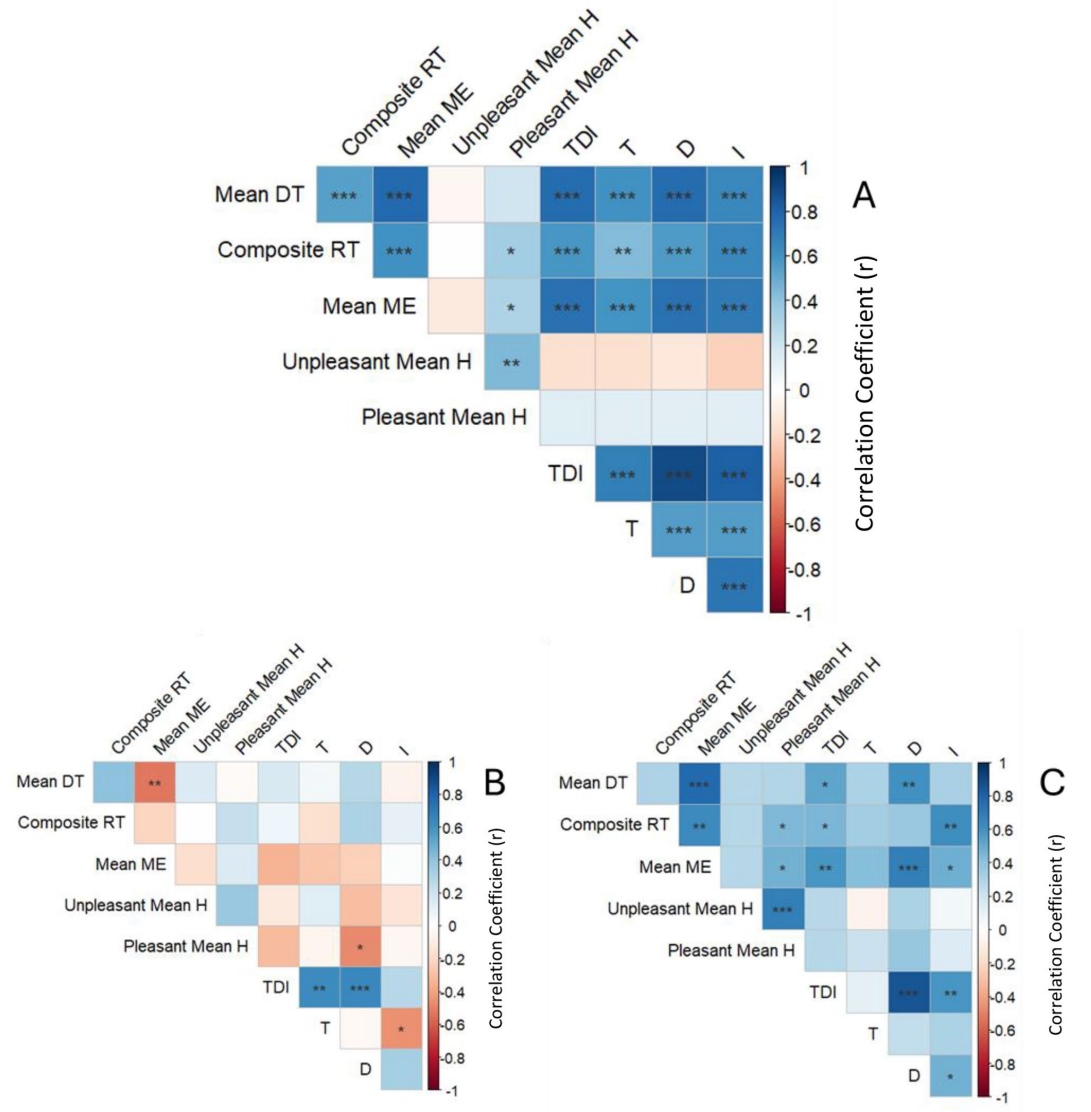
Correlation between “Sniffin’ Sticks” and Henkin olfactory test scores. **A**: Healthy controls and olfactory dysfunction patients; **B**: Healthy controls only; **C**: Olfactory dysfunction patients only; DT: detection threshold; Composite RT: sum of correct responses for recognition threshold (maximum of 4); Mean ME: mean magnitude estimation ratings for 4 odors; Unpleasant Mean H: mean hedonic ratings for Thiophene and Pyridine; Pleasant Mean H: mean hedonic ratings for Nitrobenzene and Amyl Acetate; TDI: composite “Sniffin’ Sticks” odor threshold, differentiation, and identification score; T: odor identification score; D: odor discrimination score; I: odor identification score; Box colors indicate the direction and strength of correlation (blue: positive correlation, red: negative correlation, darker colors denote stronger correlation); * *p* < 0.05, ** *p* < 0.01, *** *p* < 0.001

Mean DT, composite RT, and mean ME were all correlated with T, D, I, and TDI scores (Fig. 1). However, when checking for correlations within each group, it was only among OD patients that mean DT, composite RT, and mean ME were correlated with TDI scores, while no correlations were found for any of the Henkin subtests and TDI scores in healthy controls.

## Discussion

The test established by Henkin is able to separate patients from controls, as shown in our pilot study. However, this test tends to rely on stringent and extreme classifications for normosmia and anosmia that cluster most individuals as having hyposmia. Moreover, the actual conduct of procedure remains unclear, with several references stating different variations, and often referring to older publications with incomplete information.

Although most articles that used the Henkin test referred to “normal values” for each subtest [4], it remains unclear how these values were determined. A larger sample of patients were tested using the Henkin test [4], but this paper did not include specific normative values for each of the subtests. In this present study, most combinations of scores were not previously interpreted based on the published classification (Table 1). Furthermore, the determination of threshold relied on a conservative stepwise approach with only 2 reversals (see also [16]), and may result in variable results. The use of non-standard units of measurement along with a proportion of mean scores for 4 different tests using 4 different odors requires several calculations, which is impractical to do in a clinical setting.

The interpretation for hedonic ratings were described as follows: “If H responses which are usually positive are negative the patient exhibits... dysosmia.” [4] However, there was no specific basis for what is “usual”. Cultural differences may induce varied hedonic responses for the same odors [17, 18]. Furthermore, anosmia, especially when severe, can also result to incongruent hedonic ratings. Hs did not effectively distinguish between groups, consistent with the power of the analysis being lower compared to the other subtests, as both controls and OD patients had hedonic responses that greatly overlapped in distribution for all 4 odors (low effect size). In this respect, Hs are not so effective in distinguishing between groups, perhaps as there is no correct or incorrect response for hedonics –a generally subjective rating. Furthermore, what is pleasant or unpleasant to an individual may not necessarily change as a function of decreased quantitative OD alone. In addition, a study investigating Hs on qualitative OD also failed to show significant differences between healthy controls and patients [19]. H may also be intertwined with intensity and thus, ME may be a more reliable subtest to distinguish olfactory function between groups. Therefore, Hs appear to have limited to no use in discriminating between good and poor olfactory function.

RT has a wide variety of acceptable answers and this may have affected how well it distinguishes between groups. Items that smell differently were regarded as acceptable responses for the same odor (for AA: “paint” and “fruit” were acceptable), and may be problematic in parosmia patients. It is common for individuals to smell a given stimulus but have difficulty naming it, often when the odor is unfamiliar or if one has cognitive impairment [20, 21]. These are some reasons why the multiple forced choice method was introduced to olfactory tests: to minimize the cognitive load of testing and to improve test performance [22, 23]– as uncued answers may be difficult to score and interpret.

According to Henkin & Gouliouk, smell function can only be evaluated quantitatively and effectively using these 4 tests [5]. It may be argued that the contribution of H on olfactory assessment using the Henkin test is minimal if any. Refinement of subtests may be worth re-evaluating as having multiple subtests that do not necessarily contribute new information (i.e. H or ME) may not be beneficial in patients with diminishing attention or fatigue.

Limitations of the study include the lower power of analysis for hedonic ratings and the non-randomization of test presentation (for technical reasons “Sniffin’ Sticks” was always tested first). Future studies may explore these differences with a larger sample along with randomization of which test to conduct first.

## Conclusion

In summary, although the Henkin test pre-dated most of the psychophysical olfactory tests currently available, the lack of published studies on its reliability and validity limit its use. Present modifications to scoring and interpretation revealed that the test is able to separate between OD patients and controls. However, challenges related to the conduct of this test (conservative step-wise threshold testing, wide variability in acceptable answers for the recognition test, lack of normative values for hedonics, unclear test procedure as published in literature) remain and have already been addressed in other reliable and validated olfactory testing techniques [12, 24, 25]. Studies that used this test should be carefully interpreted. The utility of this test in its original form remains limited.
